# Multisensory Control of Multimodal Behavior: Do the Legs Know What the Tongue Is Doing?

**DOI:** 10.1371/journal.pone.0080465

**Published:** 2013-11-04

**Authors:** Jesse D. Cushman, Daniel B. Aharoni, Bernard Willers, Pascal Ravassard, Ashley Kees, Cliff Vuong, Briana Popeney, Katsushi Arisaka, Mayank R. Mehta

**Affiliations:** 1 W. M. Keck Center for Neurophysics, University of California Los Angeles, Los Angeles, California, United States of America; 2 Department of Physics and Astronomy, University of California Los Angeles, Los Angeles, California, United States of America; 3 Brain Research Institute, University of California Los Angeles, Los Angeles, California, United States of America; 4 Integrative Center for Learning and Memory, University of California Los Angeles, Los Angeles, California, United States of America; 5 Department of Neurology, University of California Los Angeles, Los Angeles, California, United States of America; 6 Department of Neurobiology, University of California Los Angeles, Los Angeles, California, United States of America; University of Nebraska Medical Center, United States of America

## Abstract

Understanding of adaptive behavior requires the precisely controlled presentation of multisensory stimuli combined with simultaneous measurement of multiple behavioral modalities. Hence, we developed a virtual reality apparatus that allows for simultaneous measurement of reward checking, a commonly used measure in associative learning paradigms, and navigational behavior, along with precisely controlled presentation of visual, auditory and reward stimuli. Rats performed a virtual spatial navigation task analogous to the Morris maze where only distal visual or auditory cues provided spatial information. Spatial navigation and reward checking maps showed experience-dependent learning and were in register for distal visual cues. However, they showed a dissociation, whereby distal auditory cues failed to support spatial navigation but did support spatially localized reward checking. These findings indicate that rats can navigate in virtual space with only distal visual cues, without significant vestibular or other sensory inputs. Furthermore, they reveal the simultaneous dissociation between two reward-driven behaviors.

## Introduction

Adaptive behavior is governed by a wide range of multisensory input stimuli, e.g. auditory and visual, and is expressed as a diverse array of behavioral modalities[1]. To fully understand how multiple behaviors combine to produce adaptive behavior it is necessary to precisely control multisensory stimuli and measure their impact simultaneously on multiple behavioral outputs. In particular, spatial learning has been studied only using motoric output of limbs, such as in the Morris water maze[2], though other behavioral modalities could also contain spatial information. Due to technical limitations simultaneous measurement of multiple spatially modulated behaviors has not been possible as studies have typically focused on a single sensory input and a single behavioral modality in a given apparatus. Approach behavior that is driven by stimuli that predict reward, such as food magazine or reward tube checking, have been extensively studied in associative learning paradigms [3]–[6] but it is unclear if reward checking shows spatial modulation as it has previously been measured only at the site of reward delivery in a conditioning apparatus. To overcome this limitation we developed a multisensory virtual reality (VR) apparatus that allows for the simultaneous measurement of both navigational behavior and reward checking, along with precisely controlled presentation of visual, auditory and reward stimuli.

Virtual reality in rodents has recently emerged as an exciting and powerful tool as it facilitates electrophysiological and optical measurements that benefit from restricting the animal’s head movement and provides precise control over sensory stimuli[7]–[13]. However, rodent VR applications thus far have been limited to the visual modality and the behavioral tasks employed are either on 1-D linear tracks[8]–[10], [12], [14] or 2-D planes of infinite size[7]. Furthermore, none of these previous approaches provided a simultaneous measure of reward checking. Utilizing our VR apparatus we developed a virtual spatial navigation task, modeled after the Morris water maze, which requires flexible navigation from multiple start locations to a hidden reward zone based on distal visual and/or auditory cues[15]. Our results show that rats readily learned spatial navigation in VR, despite the absence of significant vestibular inputs. Furthermore, reward checking was simultaneously expressed during navigation, and there was significant experience-dependent learning in both navigational and reward checking maps, however, these two spatially modulated behaviors showed a dissociation, whereby distal auditory cues failed to support spatial navigation but did support spatially modulated reward checking.

## Results

We developed a virtual reality apparatus with several major advancements that allowed for precise presentation of visual and auditory stimuli and a simultaneous measure of reward checking during navigation (see Methods S1 for details and Figure S1). The apparatus was noninvasive and did not require head-fixation, which allowed for long-term testing under low stress conditions and the expression of natural behaviors such as rearing, grooming and resting. This apparatus provided three different sensory modalities: visual, auditory and reward. The screen design allowed for a high level of immersion in the virtual environments as visual stimuli could be projected all around, above and directly adjacent to the rat. The support for the spherical treadmill was very quiet (44 dB), which further reduced stress and allowed us to present 2-D positional auditory cues using a seven speaker surround sound system with custom audio software that utilizes higher order Ambisonics (Blue Ripple’s Rapture 3D, see Figure S3). Unlike the traditional surround speaker system, our system generates an auditory soundscape, similar to the visual landscape generated by distal sources. Precisely controlled reward stimuli were also delivered in the VR maze. A capacitive touch sensor was attached to the sugar water-reward tube to measure anticipatory reward checking behavior. Thus, this technique allowed for continuous measurement of reward checking at high temporal resolution (60 Hz) at any location in a multi-sensory virtual space, and simultaneous measurement of the animal’s navigational performance and learning (the reliability of measuring 1 revolution of the sphere had a standard deviation of 0.01 revolutions). To study spatial navigation we first developed a procedure to train the rats to engage with virtual environments and constrain their navigation to finite 2-D space and avoid virtual edges (see Figure S2 and Video S1). Next, we ensured that rats were capable of utilizing our surround system to guide both navigation and reward checking to proximal auditory and visual beacons (see Figure S3).

### Rapid learning of spatial navigation in virtual space

**Figure 1:**
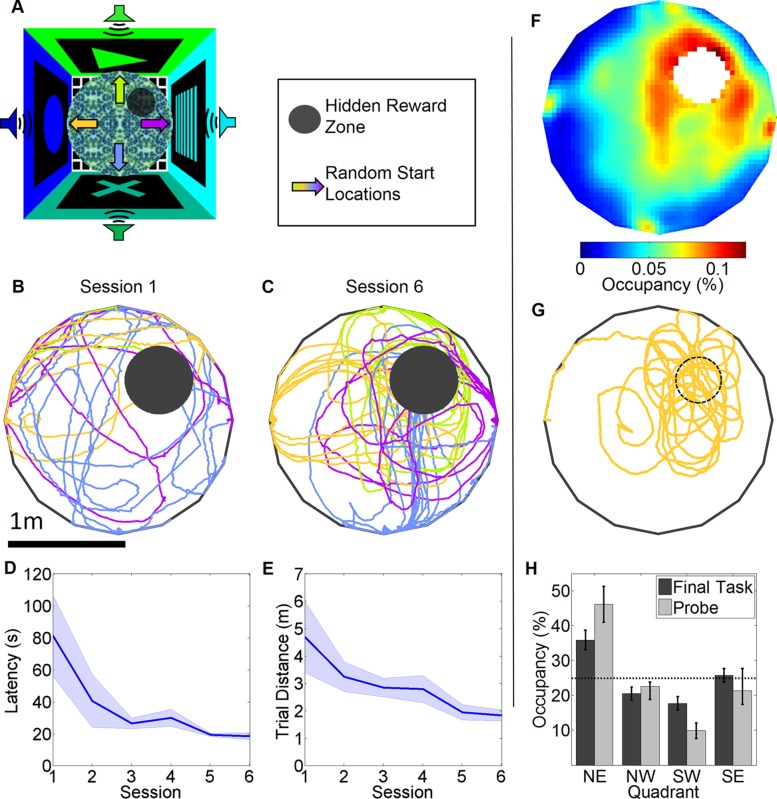
Navigational performance in the virtual audiovisual spatial navigation task. (A) Schematic of the virtual environment indicating distal auditory and visual cues and a hidden reward zone and the four start locations. (B) Example paths from a single rat from the 1^st^ session. The color of each path indicates start location, color coded from the arrows in A. (C) Example paths from the same rat from the 6^th^ session. (D) Acquisition curve of latency to reward across sessions. F(5,35)  = 4.266, p = 0.0061, Session 1 vs. Session 3: p<0.05, N =  6. (E) Acquisition curve of the distance traveled to reward across sessions. F(5,35)  = 3.00, p = 0.0296, Session 1 vs. Session 5: p<0.05. (F) 2-D histogram of mean occupancy averaged across final four task sessions of asymptotic performance with the smaller reward zone. (G) Example of a probe trial path. (H) Percentage quadrant measures for occupancy time during the final four task sessions performance and during the probe trial. Effect of quadrant: F(3,23)  = 10.15, p = 0.007, F(3,23)  = 10.9, p = 0.0005, respectively.

We trained rats in a spatial navigation task (See Video S2, Figure 1A), modeled after a standard spatial memory task, commonly referred to as the Morris water maze[15]. The rats started at one of four start locations on each trial. Their task was to navigate based on distal cues to a virtual ‘hidden reward zone’, a predetermined, unmarked place in the maze with respect to distal audio-visual cues (Figure 1B-E). Upon successful navigation they were rewarded with sugar water through the lick tube and teleported to another random start location to begin the next trial after a 2 sec inter-trial interval. Their initial search pattern was random on Session 1 but became quite accurate by Session 6 (Figure 1B vs 1C). Both the latency to find the hidden reward zone and the distance traveled to get there decreased to an asymptotic level of performance within three sessions (Figure 1D,E). To test the precision of their cognitive map, the size of the reward zone was reduced to 20 cm in radius and training was continued until a criterion of two consecutive days of 40 trials within 30 minutes. There was a clear increase in time spent around the reward zone during performance of this more difficult task (Figure 1F). To quantify this we calculated occupancy time for each quadrant (See methods). Rats spent significantly more time in the target quadrant compared to other quadrants (Figure 1H). Finally, a probe trial was conducted in which the reward zone was inactivated and rats were allowed to explore for approximately 270 seconds. Rats selectively searched at the site of the reward zone, spending significantly more time in the target quadrant (Figure 1G,H).

### Simultaneous measurement of spatially modulated reward checking during navigation

**Figure 2:**
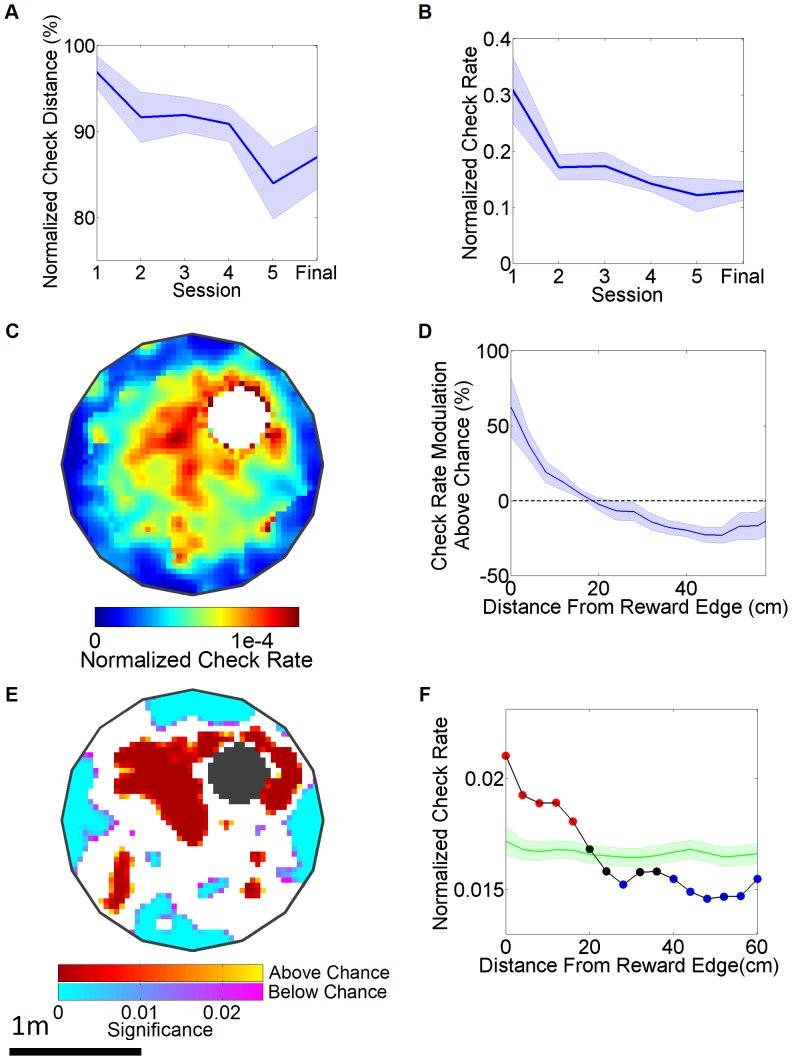
Analysis of reward checking behavior during the virtual audiovisual spatial navigation task. (A) Acquisition curve of normalized check distance across sessions and during final task performance. This is calculated as: Mean actual distance/Mean randomized distance x 100. Note that the 6^th^ day of acquisition was lost to malfunction of capacitive sensor. “Final” indicates performance average across the final four task sessions. Effect of session: F(5,35)  = 3.384, p = 0.018, p<0.05 for 1^st^ vs. 5^th^ session, N = 6. (B) Normalized check rate across sessions. Effect of session: F(5,35)  = 4.452, p = 0.0049, p<0.05 for 1^st^ vs. 4^th^ session (C) 2-D histogram of the normalized check rate averaged across rats during final task performance. (D) Check rate modulation as a function of radial distance away from the reward zone in 4 cm radial bins for final task performance. Effect of bin, F(14,89 = 9.241, p<0.0001, p<0.05 for closest bin relative to 3rd through 15th bins. (E) 2-D p-value map of a single rat’s performance during the final task performance. Red indicates regions where checking behavior was significantly modulated above chance (p<0.01) and blue indicates regions where checking behavior was significantly below chance. (F) Example of actual checking behavior (black line) as a function of radial distance from the reward zone relative to random checking behavior (green line, with shaded SE). Red dots represent points significantly above chance and blue dots represent points significantly below chance.

To understand the acquisition of reward checking behavior we calculated the average distance of each check to the reward zone normalized by the distance expected by randomly distributed licks (Referred to as normalized check distance, see Methods). This method factored out the contribution of improved navigational performance, so that spatial refinement of checking behavior could be analyzed in isolation. This analysis showed that checking shifted significantly closer to the reward zone across acquisition sessions and this was maintained during asymptotic performance with the smaller reward zone (Figure 2A). Thus, as the rats acquired the navigational component of the task they were also shifting the distribution of their checking behavior towards the reward zone. Importantly, this was above and beyond what would be expected solely by the improvement in their spatial navigation. In addition, the overall checking rate decreased (to 27% of its starting value) as they acquired the task (Figure 2B). These findings suggest that the checking-rate is modulated by the uncertainty of the reward location such that as the accuracy of checking increased its overall rate decreased.

Analysis of final asymptotic performance with the smaller reward zone showed that the reward check rate was slightly elevated in the target quadrant, but this did not reach statistical significance (Effect of quadrant: F(3,23)  = 1.234, p = 0.3319, percent check rate in target quadrant: 28%+/– 1.74 SE), which seems at odds with their spatial navigational measures showing preference for the rewarded quadrant. To analyze this further we computed the distribution of reward check rate as a function of position (Figure 2C) which showed that the check rate was elevated in the immediate vicinity of the reward zone. Since the reward check rates were very low (0.36 +/– 0.19 Hz), it was difficult to robustly estimate the significance level of reward checking in 2D bins. Hence, a 1D measure that utilized radial bins centered around the reward zone was used (Defined as: (Actual check rate in a bin – randomized check rate in that bin) / randomized check rate, see Methods). The check rate was elevated around the reward zone with the closest 4 cm bin showing a significant elevation in check rate (Figure 2D). This showed that reward checking is elevated at a very fine spatial scale just around the reward zone, but not across the entire NE quadrant. A representative example of a single rat’s performance is shown in Figure 2E and F. Checking was modulated significantly above chance in the vicinity of the reward zone (Figure 2E). Similarly, checking was significantly above chance in the first six radial bins surrounding the reward zone (Figure 2F).

### Rats rely on distal visual rather than distal auditory cues for spatial navigation

**Figure 3:**
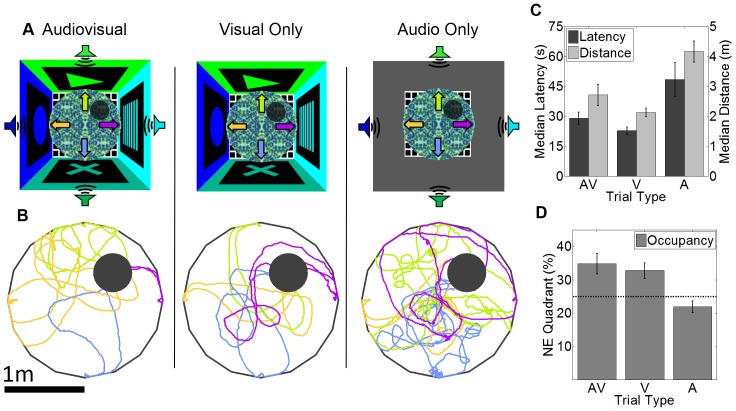
Multisensory contribution to virtual spatial navigation and reward checking. (A) Schematic of the Audiovisual (AV), Visual (V) only and Auditory (A) virtual spatial mazes. Symbols as in Figure 1. (B) Example paths for the three trial types from a single rat. The color of each path indicates start location, color coded from the arrows in A. (C) Median latency and distance to reward for each trial type. Effect of trial type, F(2,17)  = 7.555, p = 0.01, F(2,17)  = 8.911, p = 0.006, respectively. A vs. AV and V: p<0.05 for both measures, N = 6. (D) Percentage occupancy in the target quadrant for the three trial types. Effect of trial type: F(2,17)  = 13.19, p = 0.0064. A vs. AV: p = 0.013, A and V: p = 0.018.

To understand the underlying multisensory contributions to spatial navigation we systematically removed either the visual or auditory cues (Figure 3A). To ensure similarity of experience and motivation across conditions, a blocked design was used (See methods). The example paths (Figure 3B) showed that rats navigated to the reward zone for the audiovisual and visual only trials but showed a mostly random search pattern in the audio-only trials. The audio-only trials had a significantly increased latency and distance to reward, however these measures did not differ between the audiovisual and visual only trials (Figure 3C). Similarly, the percentage time spent in the target quadrant was at chance level in the audio-only trials, in contrast to the audiovisual and visual trials, which were significantly above chance (Figure 3D). These results suggest that the rats were relying almost exclusively on the distal visual cues, rather than the auditory cues, to navigate to the reward location. This test was kept as brief as possible to probe the rats’ previously acquired spatial strategy, rather than the acquisition of new strategies that would occur with further training. Unfortunately, this resulted in insufficient data to conduct a statistically reliable check rate analysis and we therefore did the following experiments to address this.

### Dissociation between spatial navigation and reward checking

**Figure 4:**
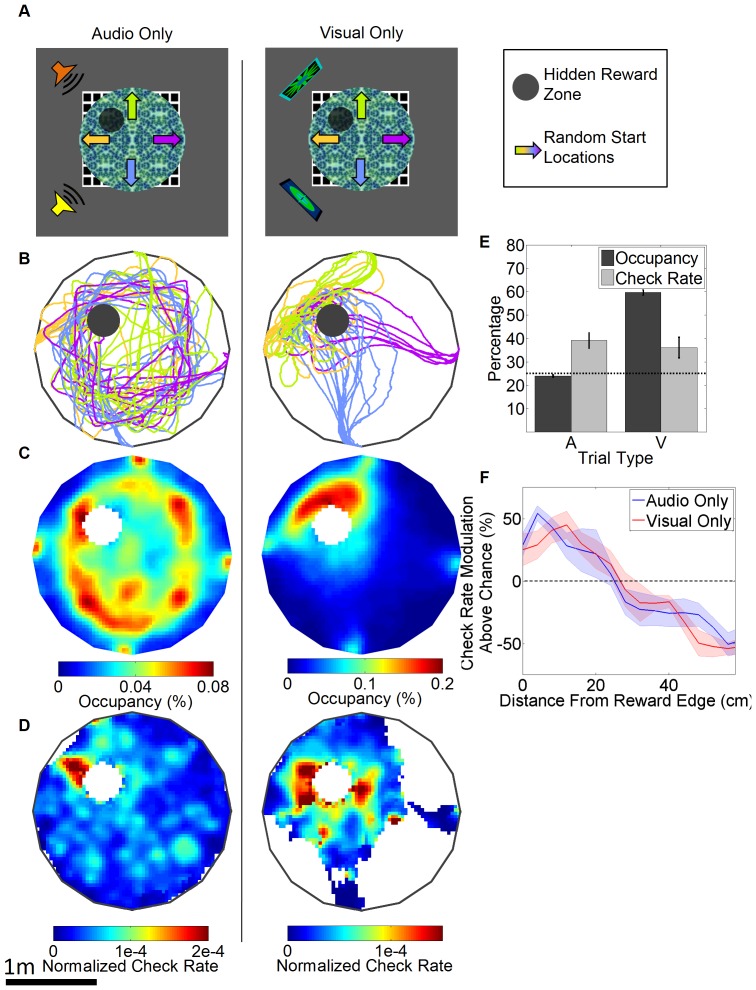
Navigational performance and reward checking during the auditory and visual virtual spatial navigation tasks. (A) Schematic of the auditory task and the visual task. Symbols as in Figure 3. (B) Example paths from each task. (C) 2-D histogram of occupancy averaged across rats over 4 sessions for each trial type. (D) 2-D histogram of the normalized check rate averaged across rats over these sessions. White areas indicate insufficient coverage. (E) Percentage of occupancy and check rate in the target quadrant for the two tasks. Two way ANOVA for effect target vs. other quadrants and auditory vs. visual trial types: Effect of quadrant F(1,8)  = 95.16, p< 0.001, Effect of task type: F(1,8)  = 2.08, p<0.001, Interaction of quadrant and task type: F(1,8)  = 2.08, p = 0.002; Effect of quadrant for auditory task: p = 0.097, target quadrant percentage occupancy: 23.9±0.5% vs. mean non-target occupancy: 25.36±0.17%, note that occupancy is slightly decreased in the target quadrant as animals entering the reward zone are teleported out; for visual task: p<0.001, target quadrant percentage occupancy: 59.6±1.15% vs. mean non-target occupancy: 13.5±0.38%, N = 5. (F) Normalized check rate as a function of distance away from the reward zone in radial bins for both trial types. Effect of distance from reward: F(14,112)  = 67.11, p<0.0001, p> 0.05 for effects of task type and interaction.

There could be two potential reasons for the rats’ inability to navigate using only the distal auditory cues in audiovisual task. First, learning about the auditory cues may be prevented, or overshadowed [16], by the presence of the visual cues. Second, the use of four distal auditory cues may have saturated or cluttered the auditory processing making it difficult to distinguish the individual sounds. To rule out both of these possibilities we trained the rats on two new spatial learning tasks. One environment contained only two distal auditory cues and the other contained only two distal visual cues (Figure 4A) in the same spatial configuration relative to the hidden reward zone (See methods). These tasks were then trained separately in sequence with the sound task first. We focused our analysis on the final four sessions of asymptotic performance in these two tasks. Latency to reward was stable across these sessions and was significantly larger in the auditory relative to the visual task (Effect of session, F(3,24)  = 0.938, p = 0.4392; Effect of task: F(1,24)  = 8.989, p = 0.0171, interaction: F(3,24)  = 1.809, p = 0.1725, latency for auditory task: 36.7±6.7 s, latency for visual task: 18.96±1.12 s, see Videos S3 and S4). The rats showed a circling strategy in the auditory task, running at a fixed distance from the visually defined edge of the table, whereas in the visual task they showed clear evidence of direct navigation to the reward quadrant from all four start locations (Figure 4B, C). The rats spent significantly more time in the target quadrant in the visual but not the auditory task (Figure 4E). These findings suggest that the rats were able to form a spatial map based on two distal visual cues, but not two distal auditory cues. Surprisingly, however, reward check rate was significantly increased in the target quadrant in not only the visual task (mean check rate: 0.20 ±0.06 Hz) but also the auditory task (mean check rate: 0.22±0.08 Hz, Figure 4D,E). Radial bin analysis of reward check rate and information content showed a very similar profile in both tasks, with a broad elevation in the vicinity of the reward zone (Figure 4F, Effect of trial type for information content: *p* = 0.78, visual: 0.149 bits, auditory: 0.152 bits). Thus, in the presence of distal auditory cues, the rats do show elevated reward check rate in the vicinity of the reward zone despite showing no evidence of spatial learning based on traditional navigational measures.

## Discussion

We developed a novel multisensory virtual reality apparatus that allowed us to present precisely controlled audiovisual and reward stimuli and simultaneously measure reward checking behavior along with virtual spatial navigation. Unlike most existing systems, our system was noninvasive which minimized stress and allowed long-term measurements under low stress conditions. Rats readily learned a virtual spatial navigation task modeled after the Morris water maze. This was an appetitive version of the commonly used aversive water maze task, where they were required to navigate to an unmarked reward zone, defined solely by the distal visual and/or auditory cues, to receive a liquid reward. Importantly, the virtual maze allowed us to ensure for the first time that there were no other cues that defined the spatial location of reward, which is difficult to achieve in the real world. This 2-D navigation task did not allow the rats to use landmark navigation strategies that are employed in typical experiments by head fixed mice on a 1-D virtual linear track[14]. Spatial navigation in virtual reality has been demonstrated in humans[17], [18], however this is the first demonstration in rodents. Our results show robust spatial navigation maps can be formed in rats in the absence of significant vestibular cues, which have been proposed to play an essential role in spatial learning by many theories [19], [20]. This argues that the mechanisms underlying spatial learning are flexible, which has important implications for the nature of these mechanisms[12], [13], [21], as well as practical implications for the use of VR in electrophysiological studies of the hippocampus[7]–[13].

The time course of spatial learning was quite rapid, comparable to that in the real world water maze, although the automated and appetitive nature of the VR apparatus allowed for far more trials to be performed within a single session. Our findings indicated that spatial navigation was based on distal visual cues rather than distal auditory cues when both modalities were trained concurrently. This was confirmed when the rats were trained in separate mazes where the distal spatial information was provided exclusively by either visual or auditory cues. We found that they were unable to form a spatial representation based on two distal auditory cues and instead adopted a stereotyped circling strategy, similar to the pattern observed in rats with hippocampal lesions[22]. Only a few studies have investigated navigation with only distal auditory cues in rodents and obtained mixed results [23], [24]. Notably, we demonstrated that rats could accurately navigate to an auditory beacon, although this performance was weaker than navigation to a visual beacon (Figure S3). In contrast, they navigated successfully with only two distal visual cues. This is consistent with the visual dominance observed in real world tasks in rodents [23], [24], as well as humans [25]–[28]. This could be explained by the weaker acuity of rats to detect the orientation of an auditory compared to visual stimulus [29]. Thus, although rats are nocturnal, under these controlled conditions, they relied overwhelmingly on visual rather than auditory cues to navigate.

During learning of the audiovisual maze reward checking progressively shifted towards the reward zone, more than what would be predicted by improved navigation alone. This provides the first evidence that reward checking too is significantly spatially modulated and its spatial accuracy improves with experience. During asymptotic performance in the audiovisual maze reward checking showed very fine spatial modulation as it was elevated only in the immediate vicinity of the reward zone. Surprisingly, reward checking was also elevated around the reward zone for the auditory maze. In fact, reward checking was equally spatially precise in the auditory and purely visual mazes (Figure 4E,F). Thus, despite no evidence of spatial learning based on navigational measures, reward checking was significantly spatially modulated by the distal auditory cues.

This remarkable dissociation argues that our auditory cues were sufficiently salient and precise to support spatially modulated behavior, which precludes more trivial explanations for their failure to support spatial navigation. More importantly, however, this argues that the nature of multisensory information processing that underlies each behavioral output shows a divergence at some point in the processing stream. This is consistent with the hypothesis that spatial navigation and reward checking, typically considered an output of associative learning[3]–[5], are driven by parallel memory systems, which operate according to their own underlying rules, or processing styles[1]. These two systems are either partially parallel, i.e. each system has access to identically processed information and uses it differently, or more fully parallel, i.e. each system may represent multisensory information in fundamentally different ways. However, it is also possible that this processing is not always in parallel, as navigation and reward checking are in register based on distal visual cues, although this would require that checking behavior switches its underlying processing mechanisms based on the modalities of the stimuli it has available.

In associative learning multisensory information is thought to be integrated into configural representations whereby conjunctions of multiple sensory elements are bound together as a unified whole, or Gestalt [30]–[32]. No single sensory element accurately defined the reward location in audio or visual navigation tasks and therefore the spatially modulated checking behavior that we observed must be driven by associations with the configuration of multisensory stimuli in the vicinity of the reward zone. Importantly, these associations can form regardless of the stimulus modality, as the less spatially informative distal auditory cues are sufficient to support conditioning. Spatial navigation, in contrast, is thought to require the formation of a spatial cognitive map that represents the environment in an allocentric metric coordinate system [19], [33]–[35]. Thus, an intriguing possibility is that reward checking is driven by a configural cognitive map, whereas navigation is driven by a spatial cognitive map. On the other hand, there may be just one cognitive map driving both navigation and reward checking but generating differential behavior due to greater metabolic costs of the former than the latter. For example, errors in computing navigational space have major energetic costs, as they will result in navigating to incorrect locations, while no such cost is incurred by erroneous reward checking. Therefore the threshold for navigational decisions ought to be high, which could produce a strong reliance on more spatially informative visual information.

The underlying neural circuitries of spatial navigation and associative learning have been under intensive investigation for some time, with the entorhinal-hippocampal system thought to be the major mediator of both spatial navigation and configural associations [1], [2], [20], [30], [36]–[41]. Our findings therefore raise the intriguing possibility that the same structure is simultaneously involved in generating the output for two behavioral systems. Future studies utilizing multisensory virtual reality combined with electrophysiological recording techniques[12] across multiple relevant brain regions will be able to probe more deeply into the underlying neural mechanisms of this parallel information processing. Furthermore, the ability to train rats in complex spatial tasks in virtual reality provides the potential to directly unify research in rodents and humans in a way that has not previously been possible [17], [18], [42]–[44].

## Materials and Methods

### Animals

Nine male Long Evans rats, approximately 3 months old at the beginning of behavioral training were used for these experiments. They were maintained on a normal, 12 hour light/dark cycle with behavioral training and testing during the light phase. The animals were food and water restricted (16–18 g of food, 25–35 ml of water per day) during behavioral training. All procedures were carried out in accordance with NIH guidelines and approved by the Animal Research Committee at UCLA.

### General Pre-training Procedures

Upon arrival, the rats were handled for 15 – 30 minutes per day. This was continued for at least 5 days at which time we began more specific pre-training procedures over 10–15 days for eventual virtual reality training. This involved three major procedures: 1) habituation to the harness for about 30 minutes per day, 2) habituation to being constrained in a harness on top of the spherical treadmill and 3) Pre-training of the reward tone-sugar water association. The latter was done in a conditioning chamber next to the VR apparatus where the rat was trained to associate the reward tone with sugar water delivery over four to five days. The reward tone was a 200 ms 1 kHz beep. It was followed by 400 ms opening of the sugar water dispensing valve, repeated 5 times.

### Virtual Spatial Navigation Task

We developed a spatial learning task modeled after the Morris water maze [15]. The virtual world (Figure 1A) consisted of a 1 meter radius circular table placed 125 cm above the floor in the center of a 4.5x4.5m room with distinct visual cues on each wall as well as four distinct complex auditory cues (North sound: Frequency sweep from 1–5 kHz repeated once a second; East sound: complex sound peaked at 2.3 kHz repeated three times a second; South sound: 10 kHz click repeated 10 times a second; West sound: complex tone containing 14–20 kHz repeated 1.5 times per second). There were no spatially informative cues on the virtual table (Figure 1A). The rat started from one of 4 random start locations, facing the wall. The northeast quadrant of the table was designated as the target quadrant. In the center of this quadrant was a 30 cm radius unmarked reward zone. Upon entry into this zone up to 5 reward pulses were dispensed. To provide visual feedback a white dot spanning the reward zone appeared upon entry. After 5 rewards were dispensed, or if the rat left the reward zone, a 2 sec blackout period was initiated and then the rat was teleported to one of the 4 random start locations facing the wall. Rats were run for a maximum of 45 minutes or 200 reward pulses. Rats were trained for 6 days of acquisition with the 30 cm radius reward zone. After this the size of the reward zone was reduced to 25 cm for one day and then down to 20 cm for several days until a criterion of two days of 200 reward pulses within 30 minutes. 24 hours after the last training day a probe trial was conducted in which the reward zone was inactivated and allowed the animal to explore for 4.5 minutes. Next, we designed a task to systematically remove either the visual or auditory cues using a blocked design with 8 trials of the audiovisual cues, 8 trials with only auditory cues and 8 trials with only visual cues. Within each block, each start location was used twice in a pseudorandom order. For this task six rats were used.

To determine if spatial learning is possible based solely on distal auditory cues, without any potential overshadowing by visual cues, we designed a task with two distal auditory cues and no distal visual cues. Two novel and distinct auditory cues were placed NW and SW of the virtual table and the 25 cm reward zone was in the center of the NW quadrant (NW sound: Complex sound centered around 8 kHz repeated three times a second; SW sound: Fluctuating sweep from 2 –7 kHz repeated 0.35 times a second, Figure 4A). This layout was based on a previous study that showed evidence of spatial navigation based solely on distal sound cues[24]. A virtual environment with the identical layout was also created except that two visual cues were used instead of the two auditory cues. Initial training occurred on a virtual table with identical dimensions to the previous audiovisual spatial navigation task. For the purely auditory task this failed to produce any evidence of spatial learning, instead indicating that the rats had adopted a general search strategy of running at a fixed distance from the edge of the table. To diminish the effectiveness of this strategy we therefore increased the radius of the virtual table to 1.2 m and decreased the reward zone to 20cm radius while leaving the relative location of the reward zone and distal auditory cues intact. In addition, due to the greater difficulty of the task the number of reward pulses was increased from 5 to 10. Training in the virtual environment with two distal visual cues followed the same procedure as training in the auditory version. The data presented in Figure 4 is from the final 4 days of performance on both of these tasks for each rat. For this task five rats that were previously trained in the spatial navigation task were used.

### Data analysis and statistical methods

A 2 cm/s speed threshold was applied to all occupancy, normalized check rate and quadrant measures to remove periods of immobility. For performance measures we calculated the median value across all trials for each rat within a session and performed subsequent analysis using these values across rats. Edge clipping was calculated by taking the total movement of the spherical treadmill into the boundaries of the virtual table divided by the total movement of the spherical treadmill during each session. Using a resampling technique we calculated the spatial distribution of random reward checking by randomly redistributing the number of checks along the rat’s path on a trial to trial basis. The normalized check distance measure was calculated by dividing the mean radial distance of reward checks by the mean radial distance of resampled reward checks (Actual mean distance / Resampled mean distance x 100). The normalized check rate was calculated by dividing the check rate in each bin by the area under the curve of the check rate across all bins within 80 cm of the reward zone for each rat. Check rate modulation above chance was calculated by subtracting the normalized mean resampled check rate in each bin from the normalized check rate in each bin then dividing by the normalized mean resampled check rate in each bin ((Actual normalized check rate - Resampled normalized check rate) / Resampled normalized check rate x 100). For the individual example in Figure 2 E,F P-values were obtained through comparison of the actual data to the resampled reward tube checking. A 4cm x 4cm bin size was used for all measures of behavior in the 2-dimensional plane. Similarly, all measures that depended on the distance from reward zone measures used a radial bin size of 4cm. Spatial distribution of reward tube check rate was computed using a 6cm (2-dimensional data) and 2.6cm (radial data) Gaussian smoothing kernel on occupancy and reward tube checking histograms. For visualization of the 2-D check rate histograms (Figure 2C,E and Figure 4D), we applied a 0.25s per bin per rat occupancy threshold. The information content of radial reward tube check rate histograms (in bits) was defined as


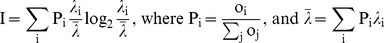


where *i* is the bin number, *P_i_* is the probability for occupancy of bin *i,* obtained from *o_j_* the occupancy in spatial bin *j*, *λ_i_* is the mean check rate for bin *i,* and 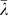 is the overall mean check rate. For quadrant analysis of occupancy time, distance traveled and reward checking during performance of the spatial learning tasks, data from inside the reward zone and the equivalent location in the other quadrants were removed prior to any calculations. The error bars in the figures represent the standard error of mean computed across all rats. For statistical analysis we utilized ANOVA, with alpha = 0.05. When justified, this was followed by Tukey post-hoc analysis and Bonferoni corrections for repeated measures comparisons.

## Supporting Information

Figure S1 **Multimodal, noninvasive, multisensory virtual reality apparatus.** (A) Cross-section schematic of VR system showing the overall frame, mirror, micro-projector, reward tube, holding mechanisms for the rat, spherical treadmill and air cushion. (B) Top-down view of the speaker arrangement surround the apparatus (C) Picture of VR system from behind while the rat is performing the virtual random foraging task. (D) A view of the virtual random foraging environment generated by the software. (E) Picture taken inside the VR from the point of view of the rat from the same point as in d. (F) A rat in VR system. Note the hinge and harness that allows a natural posture for the rat.
(TIF)

Figure S2 **Rats rapidly learn to navigate and avoid edges in a finite 2-D virtual environment.** (A) Schematics for: Session 1 in the Large Table with 5 rewards (LT5) virtual environment and Session 11 in Small Table with 1 reward (ST1) virtual environment. (B) Example of a 30 minute path from a single rat in session 1 and 11 respectively. (C) Mean Occupancy across all rats in session 1 and 11 respectively. (D) Acquisition curve of the percentage of distance traveled into the edge of the platform, referred to as edge clipping. Effect of session: F(2,32)  = 4.037, p = 0.0038, First vs. fifth session: t = 2.812, p<0.05, N = 4. (E) Acquisition curve for latency between rewards. Effect of session: F(2,32)  = 3.913, p = 0.0012, First vs. fourth session: t = 3.056, p<0.05.
(TIF)

Figure S3 **Validation of the seven speaker ambisonic surround sound system.** (A) Schematic of the Visual and Auditory beacon tasks. Arrows indicate the random starting orientations of rats on each trial. Striped circle indicates the presence of the visual beacon. Sound icon indicates presence of auditory beacon. (B) Example paths for the two trial types. The color of each path indicates the approximate starting orientation, color coded from the arrows in a. In this and all subsequent plots, areas within the reward zone are not analyzed. (C) 2-D histogram of mean occupancy averaged across all rats. (D) 2-D histogram of normalized check rate averaged across rats. White bins received insufficient sampling. (E) Median latency and distance to reward in visual (V) and auditory (A) tasks. Effect of trial type: t = 2.453, p = 0.070 and t = 3.945, p = 0.0169, respectively; A vs. AV and V, p<0.05, N = 3. (F) Sound intensity (dB) verse distance from a sound source in VR and RW. (G) Sound intensity (dB) of a sound source at different orientations in VR.
(TIF)

Methods S1 **Supplementary Methods.**
(DOCX)

Video S1 **This video shows a rat performing the virtual random foraging task.** This task was designed as their initial training for navigating in two dimensions and to avoid the edges of the virtual world.
(MP4)

Video S2 **This video shows a rat performing the virtual spatial navigation task.** The rats starts at one of four random start locations facing away from the center of the virtual platform and then must navigate to the reward location based on distal cues. After receiving reward there is a two second blackout period and then the rat is teleported to one of the four random start locations.
(MP4)

Video S3 **This video shows a rat performing the virtual spatial navigation task with only two distal auditory cues.** The rat is unable to navigate directly to the reward location. Instead he engages in a circling strategy to locate it.
(MP4)

Video S4 **This video shows a rat performing the virtual spatial navigation task with only two distal visual cues.** The rat is able to navigate directly to the reward location from any start location.
(MP4)

## References

[1] White NM, McDonald RJ (2002) Multiple parallel memory systems in the brain of the rat. Neurobiol Learn Mem 77: 125–184. 11848717 10.1006/nlme.2001.4008

[2] Morris RG, Garrud P, Rawlins JN, O'Keefe J (1982) Place navigation impaired in rats with hippocampal lesions. Nature 297: 681–683. 7088155 10.1038/297681a0

[3] Balsam PD, Drew MR, Gallistel CR (2010) Time and Associative Learning. Comp Cogn Behav Rev 5: 1–22. 21359131 10.3819/ccbr.2010.50001 PMC3045055

[4] Peterson GB, Ackilt JE, Frommer GP, Hearst ES (1972) Conditioned Approach and Contact Behavior toward Signals for Food or Brain-Stimulation Reinforcement. Science 177: 1009–1011. 17788815 10.1126/science.177.4053.1009

[5] Schultz W, Apicella P, Scarnati E, Ljungberg T (1992) Neuronal activity in monkey ventral striatum related to the expectation of reward. J Neurosci 12: 4595–4610. 1464759 10.1523/JNEUROSCI.12-12-04595.1992 PMC6575755

[6] Pan WX, Brown J, Dudman JT (2013) Neural signals of extinction in the inhibitory microcircuit of the ventral midbrain. Nat Neurosci 16: 71–78. 23222913 10.1038/nn.3283 PMC3563090

[7] Holscher C, Schnee A, Dahmen H, Setia L, Mallot HA (2005) Rats are able to navigate in virtual environments. J Exp Biol 208: 561–569. 15671344 10.1242/jeb.01371

[8] Harvey CD, Collman F, Dombeck DA, Tank DW (2009) Intracellular dynamics of hippocampal place cells during virtual navigation. Nature 461: 941–946. 19829374 10.1038/nature08499 PMC2771429

[9] Dombeck DA, Harvey CD, Tian L, Looger LL, Tank DW (2010) Functional imaging of hippocampal place cells at cellular resolution during virtual navigation. Nat Neurosci 13: 1433–1440. 20890294 10.1038/nn.2648 PMC2967725

[10] Harvey CD, Coen P, Tank DW (2012) Choice-specific sequences in parietal cortex during a virtual-navigation decision task. Nature 484: 62–68. 22419153 10.1038/nature10918 PMC3321074

[11] Keller GB, Bonhoeffer T, Hubener M (2012) Sensorimotor mismatch signals in primary visual cortex of the behaving mouse. Neuron 74: 809–815. 22681686 10.1016/j.neuron.2012.03.040

[12] Ravassard P KA, Willers B, Ho D, Aharoni DA, Cushman JD, et al. (2013) Multi-sensory control of hippocampal spatiotemporal selectivity. Science In Press. 10.1126/science.1232655 PMC4049564 23641063

[13] Chen G, King JA, Burgess N, O'Keefe J (2013) How vision and movement combine in the hippocampal place code. Proc Natl Acad Sci U S A 110: 378–383. 23256159 10.1073/pnas.1215834110 PMC3538268

[14] Youngstrom IA, Strowbridge BW (2012) Visual landmarks facilitate rodent spatial navigation in virtual reality environments. Learn Mem 19: 84–90. 22345484 10.1101/lm.023523.111 PMC3293517

[15] Morris R (1984) Developments of a water-maze procedure for studying spatial learning in the rat. J Neurosci Methods 11: 47–60. 6471907 10.1016/0165-0270(84)90007-4

[16] Mackintosh NJ (1976) Overshadowing and stimulus intensity. Animal Learning & Behavior 4: 186–192. 964444 10.3758/bf03214033

[17] Bohbot VD, Lerch J, Thorndycraft B, Iaria G, Zijdenbos AP (2007) Gray matter differences correlate with spontaneous strategies in a human virtual navigation task. Journal of Neuroscience 27: 10078–10083. 17881514 10.1523/JNEUROSCI.1763-07.2007 PMC6672675

[18] Jacobs WJ, Laurance HE, Thomas KGF (1997) Place learning in virtual space I: Acquisition, overshadowing, and transfer. Learning and Motivation 28: 521–541.

[19] McNaughton BL, Battaglia FP, Jensen O, Moser EI, et al (2006) Path integration and the neural basis of the 'cognitive map'. Nat Rev Neurosci 7: 663–678. 16858394 10.1038/nrn1932

[20] Muller RU, Poucet B, Fenton AA, Cressant A (1999) Is the hippocampus of the rat part of a specialized navigational system? Hippocampus 9: 413–422. 10495022 10.1002/(SICI)1098-1063(1999)9:4<413::AID-HIPO7>3.0.CO;2-#

[21] Eichenbaum H (2000) Hippocampus: mapping or memory? Curr Biol 10: R785–787. 11084350 10.1016/s0960-9822(00)00763-6

[22] Eichenbaum H, Stewart C, Morris RG (1990) Hippocampal representation in place learning. J Neurosci 10: 3531–3542. 2230943 10.1523/JNEUROSCI.10-11-03531.1990 PMC6570096

[23] Rossier J, Haeberli C, Schenk F (2000) Auditory cues support place navigation in rats when associated with a visual cue. Behav Brain Res 117: 209–214. 11099774 10.1016/s0166-4328(00)00293-x

[24] Sutherland RJ (1984) Place navigation by rats in a swimming pool. Canadian Journal of Psychology 38: 322–347.

[25] Howard IP, Templeton WB (1966) Human spatial orientation. London ; New York: Wiley. 533 p. p.

[26] Welch RB, Warren DH (1980) Immediate perceptual response to intersensory discrepancy. Psychol Bull 88: 638–667. 7003641

[27] Ernst MO, Bulthoff HH (2004) Merging the senses into a robust percept. Trends Cogn Sci 8: 162–169. 15050512 10.1016/j.tics.2004.02.002

[28] Talsma D, Senkowski D, Soto-Faraco S, Woldorff MG (2010) The multifaceted interplay between attention and multisensory integration. Trends Cogn Sci 14: 400–410. 20675182 10.1016/j.tics.2010.06.008 PMC3306770

[29] Heffner RS, Heffner HE (1992) Visual factors in sound localization in mammals. J Comp Neurol 317: 219–232. 1577997 10.1002/cne.903170302

[30] Fanselow MS (2000) Contextual fear, gestalt memories, and the hippocampus. Behav Brain Res 110: 73–81. 10802305 10.1016/s0166-4328(99)00186-2

[31] Rudy JW, Sutherland RJ (1989) The hippocampal formation is necessary for rats to learn and remember configural discriminations. Behav Brain Res 34: 97–109. 2765175 10.1016/s0166-4328(89)80093-2

[32] Pearce JM (2002) Evaluation and development of a connectionist theory of configural learning. Anim Learn Behav 30: 73–95. 12141138 10.3758/bf03192911

[33] Gallistel CR (1990) The organization of learning. Cambridge, MA: Bradfor/MIT Press.

[34] O'Keefe J, Nadel L (1978) The Hippocampus as a Cognitive Map. Oxford: Oxford University Press.

[35] Cheng K (1986) A purely geometric module in the rat's spatial representation. Cognition 23: 149–178. 3742991 10.1016/0010-0277(86)90041-7

[36] Dupret D, O'Neill J, Pleydell-Bouverie B, Csicsvari J (2010) The reorganization and reactivation of hippocampal maps predict spatial memory performance. Nat Neurosci 13: 995–1002. 20639874 10.1038/nn.2599 PMC2923061

[37] Hollup SA, Molden S, Donnett JG, Moser MB, Moser EI (2001) Place fields of rat hippocampal pyramidal cells and spatial learning in the watermaze. Eur J Neurosci 13: 1197–1208. 11285017 10.1046/j.0953-816x.2001.01487.x

[38] Ito R, Robbins TW, McNaughton BL, Everitt BJ (2006) Selective excitotoxic lesions of the hippocampus and basolateral amygdala have dissociable effects on appetitive cue and place conditioning based on path integration in a novel Y-maze procedure. Eur J Neurosci 23: 3071–3080. 16819997 10.1111/j.1460-9568.2006.04883.x PMC1852059

[39] Mehta MR, McNaughton BL (1997) Expansion and Shift of Hippocampal Place Fields: Evidence for Synaptic Potentiation During Behavior. New York, NY: Plenum Press.

[40] Mehta MR, Barnes CA, McNaughton BL (1997) Experience-dependent, asymmetric expansion of hippocampal place fields. Proc Natl Acad Sci U S A 94: 8918–8921. 9238078 10.1073/pnas.94.16.8918 PMC23195

[41] Mehta MR, Quirk MC, Wilson MA (2000) Experience-dependent asymmetric shape of hippocampal receptive fields. Neuron 25: 707–715. 10774737 10.1016/s0896-6273(00)81072-7

[42] Astur RS, Ortiz ML, Sutherland RJ (1998) A characterization of performance by men and women in a virtual Morris water task: a large and reliable sex difference. Behav Brain Res 93: 185–190. 9659999 10.1016/s0166-4328(98)00019-9

[43] Ekstrom AD, Bookheimer SY (2007) Spatial and temporal episodic memory retrieval recruit dissociable functional networks in the human brain. Learning & Memory 14: 645–654. 17893237 10.1101/lm.575107 PMC2044556

[44] Doeller CF, King JA, Burgess N (2008) Parallel striatal and hippocampal systems for landmarks and boundaries in spatial memory. Proc Natl Acad Sci U S A 105: 5915–5920. 18408152 10.1073/pnas.0801489105 PMC2311337

