# Supplementary figures and images for: Multisensory Control of Multimodal Behavior: Do the Legs Know What the Tongue Is Doing?
Source: PLoS One. 2013 Nov 4;8(11):e80465. doi: 10.1371/journal.pone.0080465 (PMC3817119; doi:10.1371/journal.pone.0080465)

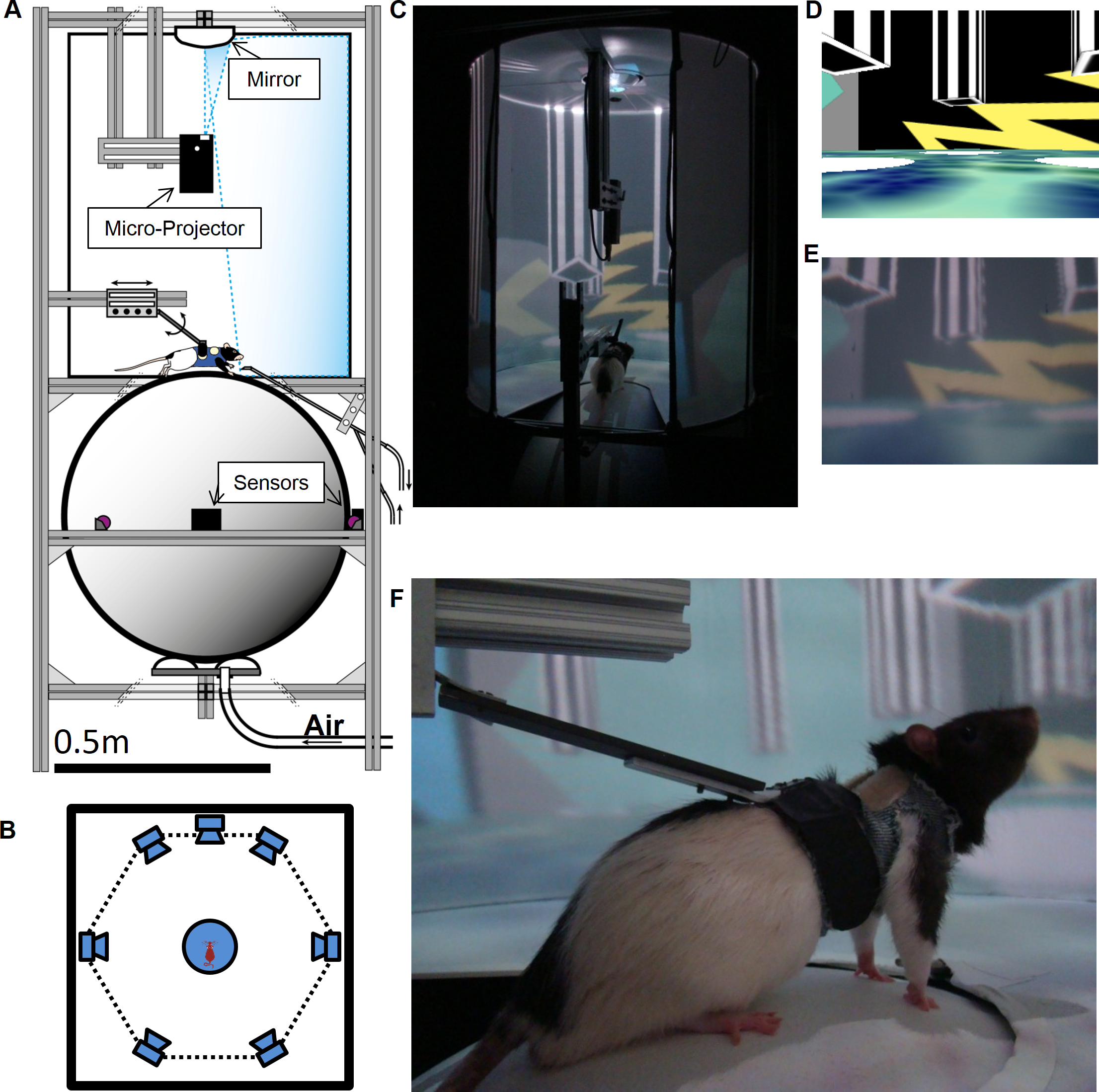

Supplement: Figure S1 — Multimodal, noninvasive, multisensory virtual reality apparatus. (A) Cross-section schematic of VR system showing the overall frame, mirror, micro-projector, reward tube, holding mechanisms for the rat, spherical treadmill and air cushion. (B) Top-down view of the speaker arrangement surround the apparatus (C) Picture of VR system from behind while the rat is performing the virtual random foraging task. (D) A view of the virtual random foraging environment generated by the software. (E) Picture taken inside the VR from the point of view of the rat from the same point as in d. (F) A rat in VR system. Note the hinge and harness that allows a natural posture for the rat. (TIF) [file pone.0080465.s001.tif]

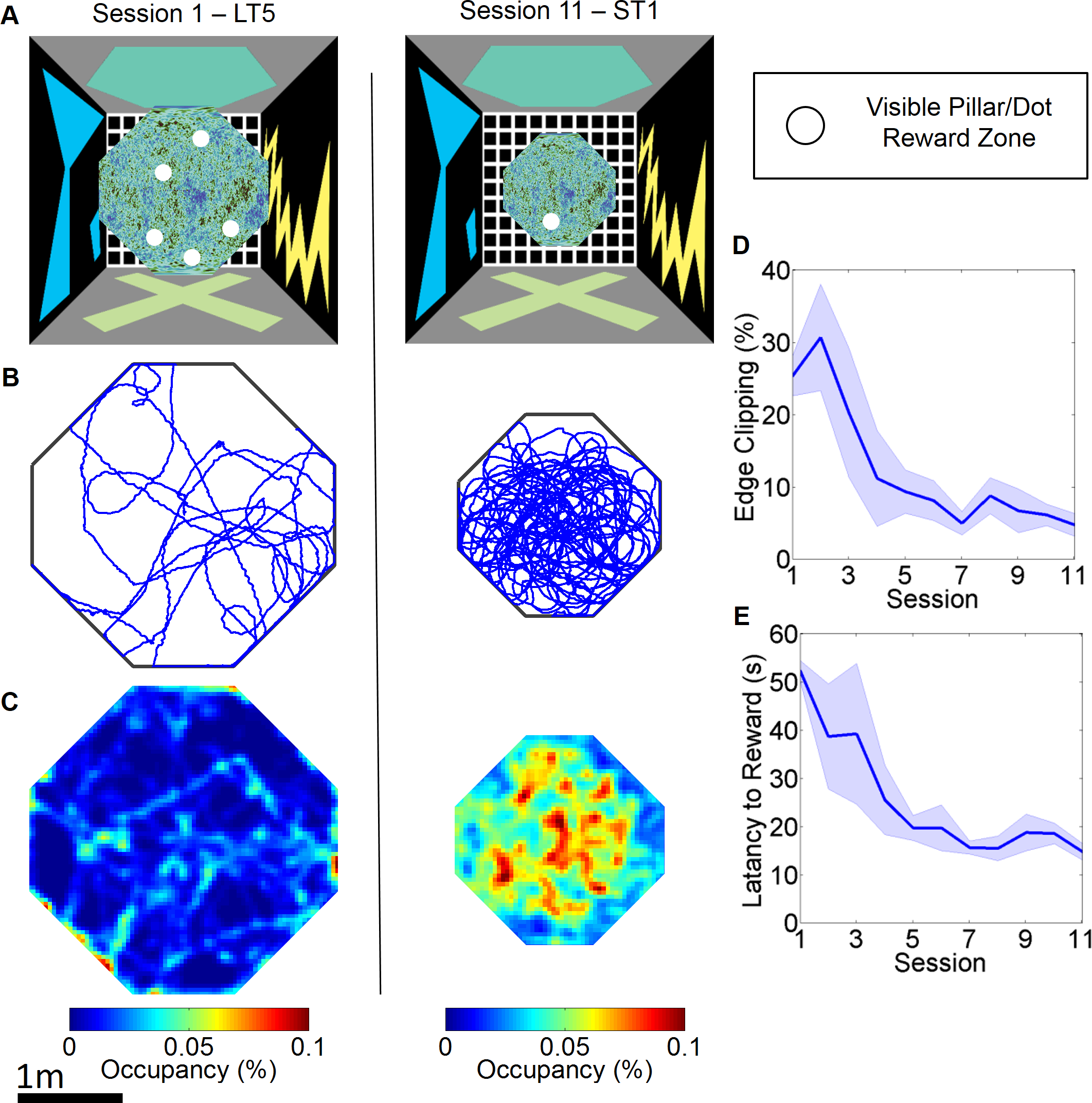

Supplement: Figure S2 — Rats rapidly learn to navigate and avoid edges in a finite 2-D virtual environment. (A) Schematics for: Session 1 in the Large Table with 5 rewards (LT5) virtual environment and Session 11 in Small Table with 1 reward (ST1) virtual environment. (B) Example of a 30 minute path from a single rat in session 1 and 11 respectively. (C) Mean Occupancy across all rats in session 1 and 11 respectively. (D) Acquisition curve of the percentage of distance traveled into the edge of the platform, referred to as edge clipping. Effect of session: F(2,32) = 4.037, p = 0.0038, First vs. fifth session: t = 2.812, p<0.05, N = 4. (E) Acquisition curve for latency between rewards. Effect of session: F(2,32) = 3.913, p = 0.0012, First vs. fourth session: t = 3.056, p<0.05. (TIF) [file pone.0080465.s002.tif]

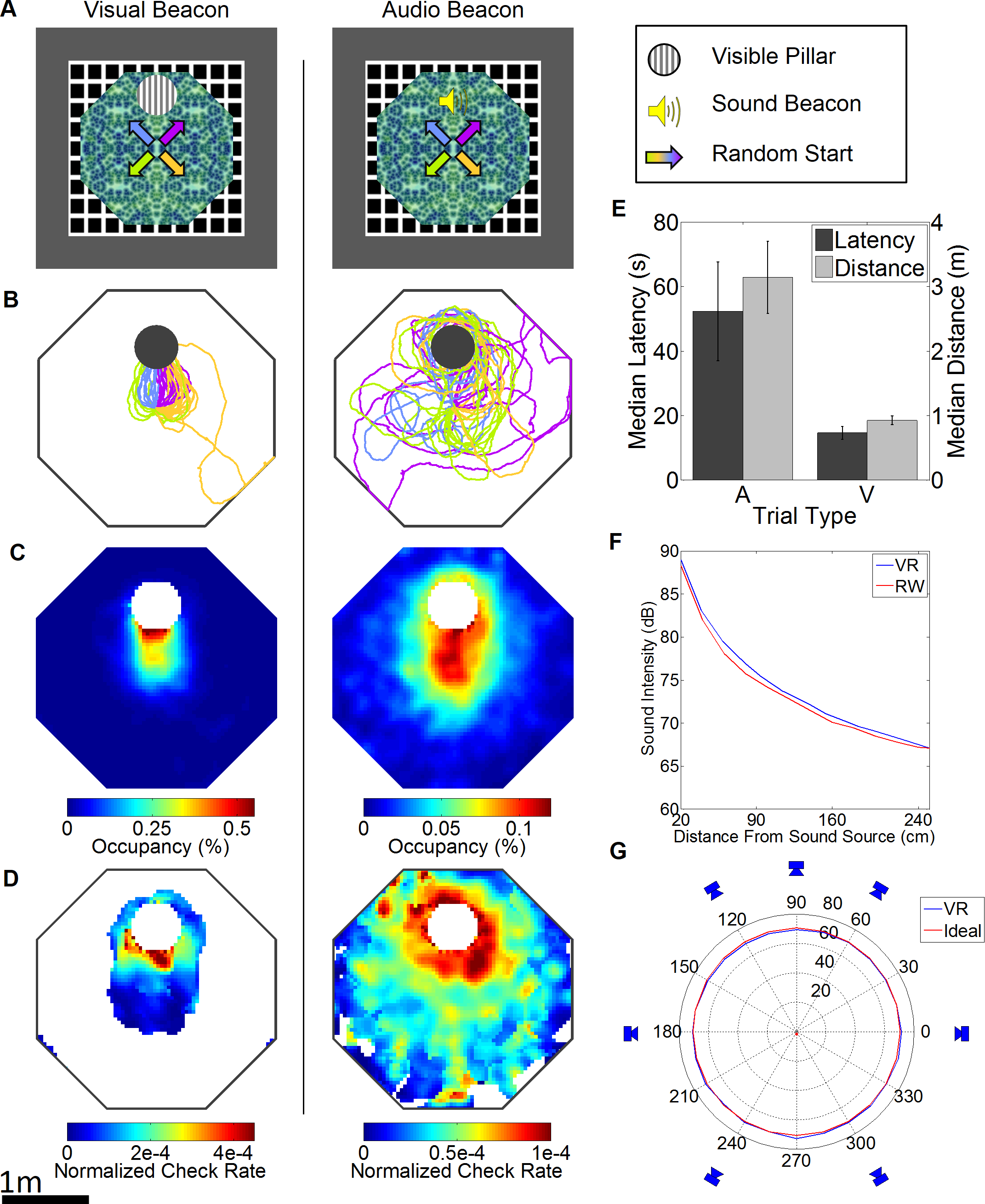

Supplement: Figure S3 — Validation of the seven speaker ambisonic surround sound system. (A) Schematic of the Visual and Auditory beacon tasks. Arrows indicate the random starting orientations of rats on each trial. Striped circle indicates the presence of the visual beacon. Sound icon indicates presence of auditory beacon. (B) Example paths for the two trial types. The color of each path indicates the approximate starting orientation, color coded from the arrows in a. In this and all subsequent plots, areas within the reward zone are not analyzed. (C) 2-D histogram of mean occupancy averaged across all rats. (D) 2-D histogram of normalized check rate averaged across rats. White bins received insufficient sampling. (E) Median latency and distance to reward in visual (V) and auditory (A) tasks. Effect of trial type: t = 2.453, p = 0.070 and t = 3.945, p = 0.0169, respectively; A vs. AV and V, p<0.05, N = 3. (F) Sound intensity (dB) verse distance from a sound source in VR and RW. (G) Sound intensity (dB) of a sound source at different orientations in VR. (TIF) [file pone.0080465.s003.tif]
