# Supplementary material for: Multisensory Control of Multimodal Behavior: Do the Legs Know What the Tongue Is Doing?
Source: PLoS One. 2013 Nov 4;8(11):e80465. doi: 10.1371/journal.pone.0080465 (PMC3817119; doi:10.1371/journal.pone.0080465)
Supplement: Methods S1 — Supplementary Methods. (DOCX) [file pone.0080465.s004.docx]

**Supporting Information**

**Supplementary Methods**

**Virtual Reality Apparatus**

**The Spherical Treadmill.** The rats ran on a 24” diameter Styrofoam sphere (Figure S1A), hollowed out and coated with Foam Coat (Special Effects Supply Corporation, North Salt Lake, Utah). The sphere rested on an air cushion allowing for low friction rotation about all three axes. The cushion was made of a thin Polyethylene foam sheet attached across a 9” diameter plastic disk. 3 standard cubic feet per minute of air inflated the cushion and flowed out of six ¼” holes cut into the foam sheet. The cushion achieved an acoustic noise level of less than 44dB, making it ideal for use with virtual auditory cues. The motion of the sphere was detected by two orthogonally positioned laser sensors (ADNS-9500, Avago) connected to a microcontroller, which merged the sensor data and sent it to the VR software (Figure S1B). With direct control over the laser sensors we achieved true one-to-one mapping of the rat’s movements into the virtual environment. The reliability of measuring 1 revolution of the sphere had a standard deviation of 0.01 revolutions. The rat was placed in a hinged harness which attached to a support frame above the treadmill (Figure S1F), which allowed a full range of natural movements of the rat including grooming, rearing, and head scanning, while restricting their spatial position.

**Visual Stimuli.** A micro-projector (MPro160, 3M, Figure S1A) was used to project pre-distorted images onto a cylindrical screen by means of a custom made convex mirror. The screen was a 75cm tall, 68cm diameter white fabric cylinder (Figure S1A,C, Hollywood Lamp & Shade, Hollywood, CA). Based on the geometry of the screen, the location of the rat, and the properties of the projector we calculated the curvature of the convex mirror to generate any predefined azimuthally symmetric pixel distribution. Our mirror construction algorithm generated machining files, vertex and normal files for the VR software, and a mapping file of the VR software’s cube map. The distortions due to the mirror’s curvature and screen shape cancelled the pre-distortion of the projected image to produce a non-distorted virtual world from the rat’s point of view (Figure S1D,E). With the small shadow of the micro-projector and custom screen and mirror, visual stimuli can be projected all around the rat and within 1cm of the rat on the platform placed just above the ball in front of the rat’s feet. Unlike existing rodent virtual reality designs, this allowed for visual stimulation in the lower half of the visual field, which is known to play an important role in visual discriminations[[1](#_ENREF_1)] and modulation of place cell firing.

**Auditory Stimuli.** 7 speakers (SS-B1000, Sony) were placed outside the screen in a hexagonal + center speaker distribution, all in an acoustically shielded, 3x3m room (Figure S1B). Higher Order Ambisonic sound was generated using Blue Ripple’s Rapture3D openAL libraries. An HD audio/video card (Radeon 5670, VisionTek) was used to output 7 audio channels over HDMI to an AV Receiver (STRDH520, Sony) which was then sent to the speakers. This hardware, in conjunction with our custom software, can generate realistic 2-dimensional spatial auditory stimuli by reconstructing the sound field in the center of the speaker array. In addition, the use of the thin fabric screen ensured that there were no distortions in the acoustic stimuli.

**Reward Delivery and Checking System.** A software driven solenoid (003-0141-900, Parker) placed outside the experimental room controlled the delivery of sugar water (10% sucrose) through a stainless steel tube mounted in front of the rat. A capacitive touch circuit (AT42QT1010, Atmel) was connected to the delivery tube that detected when the rat contacted the reward tube, providing a temporally precise (~16ms) estimate of reward checking and reward consumption. The capacitive touch sensor was read by the same microcontroller that also read the laser sensors.

**Software.** Our VR software package was custom-built in C++ using the Ogre 3D graphics engine and Open Audio Library. Virtual worlds were constructed using xml formatted text files. World type, track boundaries, reward types, reward locations, teleportation, event triggers, and visual and auditory cues are all defined in the xml file allowing easy creation and modification of environments. Pre-distortion of the visual world was handled through custom mapping files which take into account the screen geometry, mirror curvature, and pixel location. Spatial and non-spatial auditory stimuli were handled through OpenAL and capable of driving any number of speakers in any arbitrary speaker configuration. The software connected through USB to the reward delivery system, motion sensors, and capacitive touch sensor. 3 axis rotational position of the treadmill, virtual position, view angle, timestamps, and trigger events were saved. Event triggers include reward dispensing, teleports, cue hiding/revealing, and any other event defined in the xml file.

**Behavioral Procedures**

**Virtual Random Foraging Task.** We first developed a shaping procedure designed to train rats to navigate in virtual environments based on navigating to randomly dispersed visual beacons that signify reward. The rat started in the middle of an octagonal table placed in a virtual square room (3 x 3 m, Figure S2A, Supplementary Video 1). The table was 150 cm above the floor of the room, indicated by a black and white grid pattern. The table consisted of a green pattern designed to provide optic flow feedback without providing any spatial information. The visual beacons were 10 cm wide pillars with vertical black and white stripes, suspended above the virtual table. Immediately below the visual beacons were 20 cm radius white dots. When the rat entered these white dots the reward tone played and sugar water was dispensed for 400 ms. As long as the rat stayed in the dot he could receive up to 5 tone-sugar water pulses per dot. When the rat received all 5 rewards or it exited the white dot the reward was inactivated, the pillar and dot disappeared and the reward was available under new pillars/dot placed at a random location. A shaping procedure was used, where the first virtual environment consisted of a large table (1.5 m radius) with five pillars at any given time (abbreviated LT5). The rats were advanced to progressively more difficult tasks by reducing the number of pillars and the size of the table. The progression was from a Large Table with 5 pillars (LT5) 🡪 Large Table with 3 pillars (LT3) 🡪 Small Table with 2 pillars (1 m radius, ST2) 🡪 Small Table with 1 pillar (ST1). The rats were allowed to run for up to 35 minutes or until 200 rewards had been dispensed. The criterion for advancement to the next stage was 200 rewards delivered within 30 minutes. As shown by both the example path (Figure S2B) and the occupancy (Figure S2C) rats learned to effectively navigate within this virtual environment. Rats rapidly learned to quickly maneuver away from and then avoid the edges of the virtual table, reaching an asymptotic level of performance within five sessions (Figure S2D). In addition, they rapidly learned to navigate to the pillars, reaching an asymptotic level of rewards per minute within four sessions (Figure S2E). Thus, the majority of improvement in this virtual foraging task occurs during the first four days of experience with the large virtual table and five random reward zones (LT5).

Notably, the edges of the virtual world were defined solely by visual cues and did not provide somatosensory stimulation that typically defines boundaries in the real world. Thus, this task replicates previous findings that rodents can navigate to virtual visual beacons[[2](#_ENREF_2)] or landmarks[[3](#_ENREF_3)] and significantly extends these by showing that rats readily learned to respect virtual edges and can therefore be constrained within finite 2-D virtual environments.

**Supplementary Results**

**Behavioral validation of the surround sound system using an audiovisual beacon navigation task**

To validate that rats were capable of responding to our positional ambisonic surround system we developed abeacon navigation task. Three rats were trained to navigate to proximal auditory and visual beacon cues (Figure S3A). Rats learned to navigate to both auditory and visual beacons and showed greater percentage occupancy of the quadrant containing the beacon (Figure S3B-D, effect of quadrant for visual trials: F(3,6) = 45.604, p < 0.001, mean beacon quadrant occupancy: 51.9 ± 3.25%; effect of quadrant for auditory trial types: F(3,6) = 5.891, p = 0.032, mean beacon quadrant occupancy: 40 ± 4.99%). However, their navigational performance to the auditory beacon was significantly worse than the other two conditions. Occupancy was far more spatially dispersed on the auditory trials (Figure S3C) and both latency and distance to reach the beacon were longer relative visual trials (Figure S3E). Thus, although the auditory beacon can support navigational performance, it is much less effective than the visual beacon. This could be explained by the weaker acuity of rats to detect the orientation of an auditory compared to visual stimulus and the hypothesis that primary function of auditory localization is to bring the source of the stimulus into the center of the visual field[[4](#_ENREF_4)]. In addition to these behavioral measures we also confirmed that our surround sound system reliably replicated features of sound in the real world. The sound intensity dropped off as a function of virtual distance to the sound source in a nearly identical fashion as that measured in the real world (Figure S3F). In addition, the sound intensity at a fixed virtual distance was constant regardless of the virtual direction of the sound (Figure S3G). Thus, our ambisonic surround system reliably replicates the acoustic qualities of real world sound and is able to support beacon navigation and localized reward checking behavior.

**Supplementary References**

1. Minini L, Jeffery KJ (2006) Do rats use shape to solve "shape discriminations"? Learn Mem 13: 287-297.

2. Holscher C, Schnee A, Dahmen H, Setia L, Mallot HA (2005) Rats are able to navigate in virtual environments. J Exp Biol 208: 561-569.

3. Youngstrom IA, Strowbridge BW (2012) Visual landmarks facilitate rodent spatial navigation in virtual reality environments. Learn Mem 19: 84-90.

4. Heffner RS, Heffner HE (1992) Visual factors in sound localization in mammals. J Comp Neurol 317: 219-232.

**Supporting Figure Legends**

**Supplementary Figure 1.** Multimodal, noninvasive, multisensory virtual reality apparatus. (A) Cross-section schematic of VR system showing the overall frame, mirror, micro-projector, reward tube, holding mechanisms for the rat, spherical treadmill and air cushion. (B) Top-down view of the speaker arrangement surround the apparatus (C) Picture of VR system from behind while the rat is performing the virtual random foraging task. (D) A view of the virtual random foraging environment generated by the software. (E) Picture taken inside the VR from the point of view of the rat from the same point as in d. (F) A rat in VR system. Note the hinge and harness that allows a natural posture for the rat.

**Supplementary Figure 2.** Rats rapidly learn to navigate and avoid edges in a finite 2-D virtual environment. (A) Schematics for: Session 1 in the Large Table with 5 rewards (LT5) virtual environment and Session 11 in Small Table with 1 reward (ST1) virtual environment. (B) Example of a 30 minute path from a single rat in session 1 and 11 respectively. (C) Mean Occupancy across all rats in session 1 and 11 respectively. (D) Acquisition curve of the percentage of distance traveled into the edge of the platform, referred to as edge clipping. Effect of session: F(2,32) = 4.037, p = 0.0038, First vs. fifth session: t = 2.812, p < 0.05, N = 4. (E) Acquisition curve for latency between rewards. Effect of session: F(2,32) = 3.913, p = 0.0012, First vs. fourth session: t = 3.056, p < 0.05.

**Supplementary Figure 3.** Validation of the seven speaker ambisonic surround sound system (A) Schematic of the Visual and Auditory beacon tasks. Arrows indicate the random starting orientations of rats on each trial. Striped circle indicates the presence of the visual beacon. Sound icon indicates presence of auditory beacon. (B) Example paths for the two trial types. The color of each path indicates the approximate starting orientation, color coded from the arrows in a. In this and all subsequent plots, areas within the reward zone are not analyzed. (C) 2-D histogram of mean occupancy averaged across all rats. (D) 2-D histogram of normalized check rate averaged across rats. White bins received insufficient sampling. (E) Median latency and distance to reward in visual (V) and auditory (A) tasks. Effect of trial type: t = 2.453, p = 0.070 and t = 3.945, p = 0.0169, respectively; A vs. AV and V, p < 0.05, N = 3. (F) Sound intensity (dB) verse distance from a sound source in VR and RW. (G) Sound intensity (dB) of a sound source at different orientations in VR.

**Supplementary Video 1.** This video shows a rat performing the virtual random foraging task. This task was designed as their initial training for navigating in two dimensions and to avoid the edges of the virtual world.

**Supplementary Video 2.** This video shows a rat performing the virtual spatial navigation task. The rats starts at one of four random start locations facing away from the center of the virtual platform and then must navigate to the reward location based on distal cues. After receiving reward there is a two second blackout period and then the rat is teleported to one of the four random start locations.

**Supplementary Video 3.** This video shows a rat performing the virtual spatial navigation task with only two distal auditory cues. The rat is unable to navigate directly to the reward location. Instead he engages in a circling strategy to locate it.

**Supplementary Video 4.** This video shows a rat performing the virtual spatial navigation task with only two distal visual cues. The rat is able to navigate directly to the reward location from any start location.
